# Inhibitory Effects and Underlying Mechanism of 7-Hydroxyflavone Phosphate Ester in HeLa Cells

**DOI:** 10.1371/journal.pone.0036652

**Published:** 2012-05-04

**Authors:** Ting Zhang, Jiang Du, Liguo Liu, Xiaolan Chen, Fang Yang, Qi Jin

**Affiliations:** 1 MOH Key Laboratory of Systems Biology of Pathogens, Institute of Pathogen Biology, Chinese Academy of Medical Sciences & Peking Union Medical College, Beijing, People's Republic of China; 2 Key Laboratory of Chemical Biology, Department of Chemistry, Zhengzhou University, Zhengzhou, People's Republic of China; King Faisal Specialist Hospital & Research Center, Saudi Arabia

## Abstract

Chrysin and its phosphate ester have previously been shown to inhibit cell proliferation and induce apoptosis in Hela cells; however, the underlying mechanism remains to be characterized. In the present study, we therefore synthesized diethyl flavon-7-yl phosphate (FP, C_19_H_19_O_6_P) by a simplified Atheron-Todd reaction, and explored its anti-tumor characteristics and mechanisms. Cell proliferation, cell cycle progression and apoptosis were measured by MTS, flow cytometry and terminal deoxynucleotidyl transferase dUTP nick end labeling techniques, respectively in human cervical cancer HeLa cells treated with 7-hydroxyflavone (HF) and FP. p21, proliferating cell nuclear antigen (PCNA) and cAMP levels in Hela cells were analyzed by western blot and radioimmunoassay. Both HF and FP inhibited proliferation and induced apoptosis in HeLa cells via induction of PCNA/p21 expression, cleaved caspase-3/poly (ADP-ribose) polymerase (PARP)-1, elevation of cAMP levels, and cell cycle arrest with accumulation of cells in the G0/G1 fraction. The effects of FP were more potent than those of HF. The interactions of FP with Ca^2+^-calmodulin (CaM) and Ca^2+^-CaM-phosphodiesterase (PDE)1 were explored by electrospray ionization-mass spectrometry and fluorescence spectra. FP, but not HF, formed non-covalent complexes with Ca^2+^-CaM-PDE1, indicating that FP is an inhibitor of PDE1, and resulting in elevated cellular cAMP levels. It is possible that the elevated cAMP levels inhibit growth and induce apoptosis in Hela cells through induction of p21 and cleaved caspase-3/PARP-1 expression, and causing down-regulation of PCNA and cell cycle arrest with accumulation of cells in the G0/G1 and G2/M fractions. In conclusion, FP was shown to be a Ca^2+^-CaM-PDE inhibitor, which might account for its underlying anti-cancer mechanism in HeLa cells. These observations clearly demonstrate the special roles of phosphorylated flavonoids in biological processes, and suggest that FP might represent a potential new drug for the therapy of human cervical carcinoma.

## Introduction

Among the variety of natural products, flavonoids have always attracted considerable interest because of their potential beneficial effects on human health and their widespread availability in fruits, vegetables, herbs and some beverages [1], [2]. Most flavonoids have demonstrated anti-tumor properties including anti-proliferation, cell cycle arrest in G0/G1 or G2/M, and induction of differentiation and apoptosis in various cell lines [3]–[5].

A large number of phosphorus compounds possess P-O bond(s) as phosphate esters (e.g., DNA, RNA, ATP, phospholipids, etc.) and these esters of phosphoric acid play a vital role in many biological processes [6], [7]. They appear to be synthesized and undergo interconversion with great ease in living organisms [8]. Our previous studies showed that phosphorylated flavonoids possess relatively stronger binding affinities towards proteins such as myoglobin, insulin, and lysozyme and more easily form non-covalent compounds with them, compared to non-phosphorylated forms [9], [10]. As part of a screening program, we previously reported that phosphorylated chrysin did indeed exhibit stronger activity against HeLa tumor cells *in vitro* than non-phosphorylated chrysin [11]. These positive biomedical effects are mostly attributed to the potential of flavonoids to act as esters of phosphoric acid, but the underlying mechanism remains unclear. To explore these mechanisms, the phosphate ester (FP, C_19_H_19_O_6_P) of 7-hydroxyflavone (HF) was synthesized via a simplified Atheron-Todd reaction. It is an established fact that biochemical activities depend on the individual structure, and each compound needs to be studied systematically to assess its individual biological potency. In this study, we explored the anti-tumor characteristics of HF/FP with only one hydroxyl/phosphorylated structure in the flavone subgroup. The effects of these compounds on proliferation and apoptosis in HeLa cells could thus be assessed and compared.

MTS, flow cytometry, proliferating cell nuclear antigen (PCNA) immunohistochemistry and terminal deoxynucleotidyl transferase dUTP nick end labeling (TUNEL) techniques were used to gain an insight into the mechanisms of growth inhibition, cell proliferation, cell cycle progression, and apoptosis, respectively. Apoptosis was also determined by FACScanto II analysis. Semi-quantitative western immunoblotting was performed to assess the effects of HF/FP on protein expression levels. Alterations in cAMP levels were measured by radioimmunoassay (RIA). In addition, Ca^2+^-CaM-PDE complex inhibition was analyzed *in vitro* to provide detailed information about the possible mechanisms responsible for the anti-cancer activities. Moreover, we investigated the interactions between HF and FP, and the Ca^2+^-CaM-PDE enzyme system using fluorescence spectrometry to gain evidence for this mechanism in living systems. The results of this study may have important implications for the use of FP as a potent new agent for cancer prevention, as well as for other pharmacological and toxicological uses.

## Results

### Growth inhibitory effects of FP and HF in Hela cells

**Figure 1:**
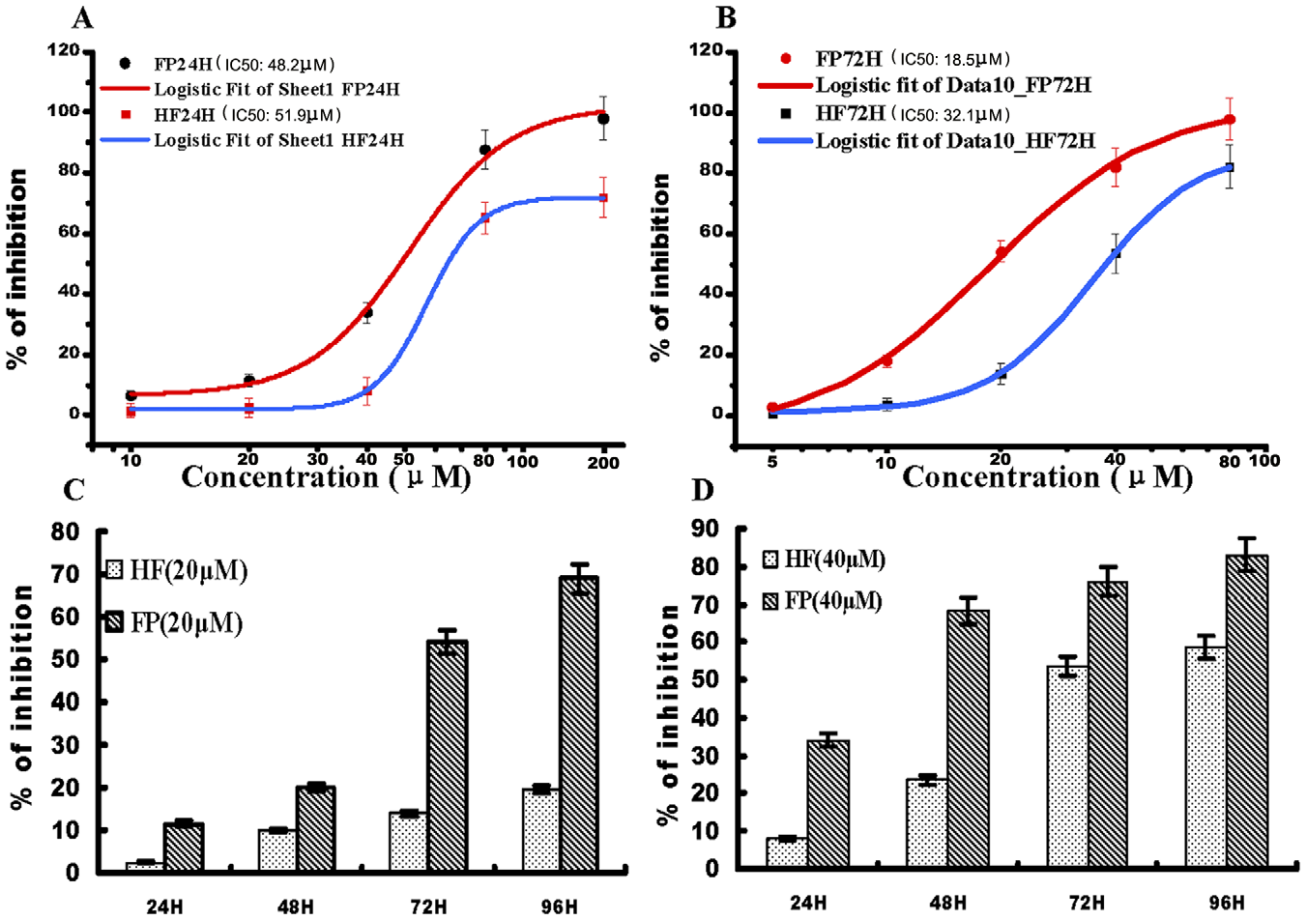
Effects of test compounds on cell growth inhibition. A) Effects of test compounds at different concentrations on HeLa cells following treatment for 24 h. IC_50_ values for FP and HF at 24 h were 46.8 and 56.9 µM, respectively. B) Effects of test compounds at different concentrations on HeLa cells following treatment for 72 h. IC_50_ values for FP and HF at 72 h were 18.5 and 32.1 µM, respectively. C) Effects of 20 µM HF and FP on cell viability following treatment for 24, 48, 72 and 96 h. D) Effects of 40 µM HF and FP on cell viability following treatment for 24, 48, 72 and 96 h. All data points represent mean ± SEM values.

Cell growth was inhibited by 5, 10, 20, 40, 60 or 80 µM HF and FP for 24 or 72 h in dose-dependent manners (Figure 1 A and B). The estimated IC_50_ values at 24 h were 51.9 µM for HF and 48.2 µM for FP, and those at 72 h were 32.1 µM for HF and 18.5 µM for FP. Cultured human HeLa cells were treated with HF and FP at concentrations of 20 and 40 µM for 24, 48, 72 and 96 h. HF and FP caused marked reductions in cell viability in time-dependent manners, compared to the control group, as shown by MTS assay. FP had a more potent effect on cell viability than HF.

### Effects of FP and HF on cell cycle distribution

**Figure 2:**
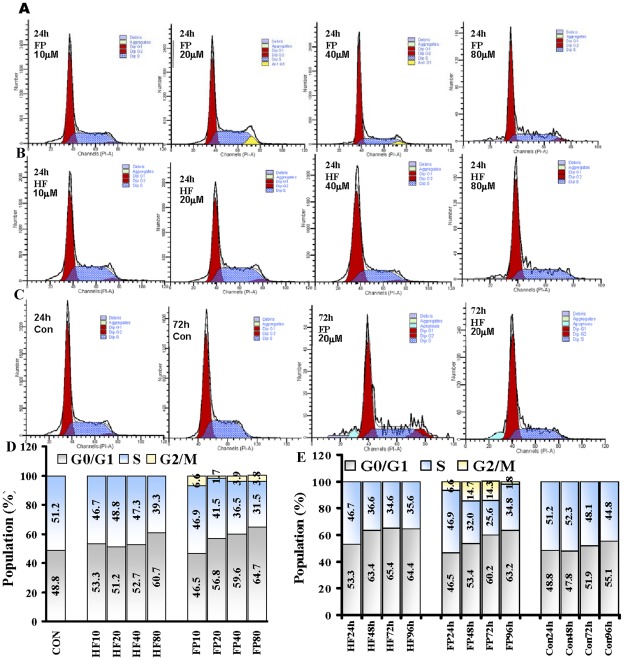
Effects of FP/HF on cell cycle distribution. HeLa cells were treated with different concentrations of FP/HF for 24, 48, 72 and 96 h. Cells were harvested and stained with propidium iodide. Cell cycle distribution was analyzed using a BD FACSCanto II flow cytometer. The distributions of cells in G0/G1, S, and G2/M phases were determined using Modfit software. A) Representative cell cycle distributions after treatment with FP at 10, 20, 40 and 80 µM for 24 h. B) Representative cell cycle distributions after treatment with HF at 10, 20, 40 and 80 µM for 24 h. C) Representative cell cycle distributions after treatment with 20 µM FP/HF at 72 h and control at 24 and 72 h. D) G0/G1, S and G2/M population percentages in each group following treatment with 10, 20, 40 and 80 µM HF or FP for 24 h. E) G0/G1, S and G2/M population percentages in each group following treatment with 20 µM HF or FP and control group for 24, 48, 72 and 96 h.

Cell cycle analysis using propidium iodide (PI) staining and flow cytometry was used to determine the effects of HF and FP on cell cycle perturbation. The cell cycle distributions of HeLa cells treated with FP and HF 10, 20, 40 and 80 µM at various time points are shown in Figure 2. Both FP (Figure 2A) and HF (Figure 2B) significantly altered cell cycle progression. They induced cell-growth arrest in HeLa cells in a dose-dependent fashion at 24 h, and 20 µM FP and HF also arrested the cell cycle in time-dependent manners, compared to the control group (Figure 2 C, D and E). As shown in Figure 2D, >40 µM FP or 80 µM HF significantly increased the percentage of HeLa cells in G1 phase, accompanied by a decrease in the population in S phase, compared to the control group, suggesting that the cell cycle was arrested at G0/G1 phase. There was a significant increase in the cell population in G2/M phase (Figure 2 D and E) following treatment with FP, as well as a marked increase in the population in G0/G1 phase and a compensatory decrease in the population in S phase. These data suggest that HF induces cell cycle arrest in G0/G1 phase, while FP induces cell cycle arrest in both G0/G1 and G2/M phases.

### FP- and HF-induced apoptosis

**Figure 3:**
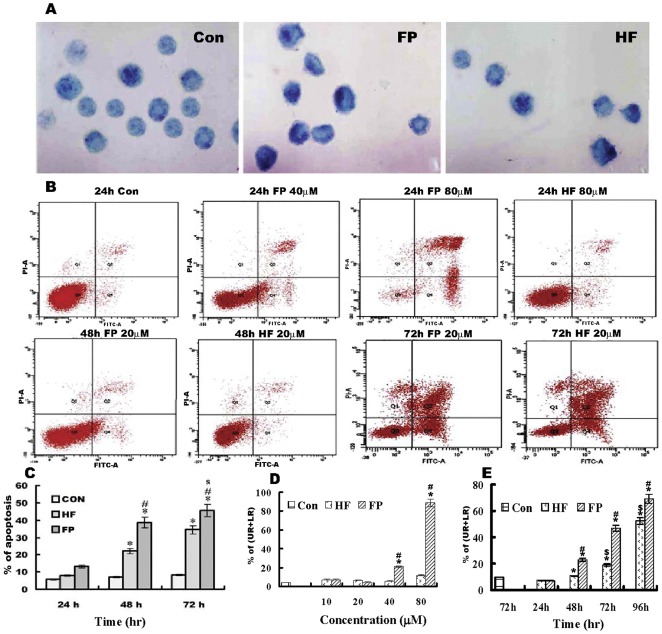
Effects of FP/HF on cell apoptosis. A) TUNEL staining for apoptosis in HeLa cells after treatment for 48 h in control, FP and HF groups; methyl green counter-stained, 1,000×. Control group, few apoptotic cells were found; FP group, more apoptotic cells were found. B) Quantitative analysis of apoptotic cells using Annexin V-FITC/PI double staining in exponentially growing HeLa cells. C) Apoptosis percentages in the different groups detected using the TUNEL method at 24, 48 and 72 h. D) Apoptosis percentages (upper right (UR)+lower right (LR)) in each group following treatment with 10, 20, 40 and 80 µM HF or FP detected by flow cytometry at 24 h. E) Apoptosis percentages (UR+LR) in each group analyzed by flow cytometry at 24, 48, 72 and 96 h. Flow cytometry was performed on 5×10^5^ cells and the percentages of apoptotic, live, and dead cells were measured. Upper left (UL; Annexin V-FITC^−^/PI^+^) represents dead cells, upper right (UR; Annexin V-FITC^+^/PI^+^) represents late apoptotic plus necrotic cells, lower left (LL; Annexin V-FITC^−^/PI^−^) represents live cells, and lower right (LR; Annexin V-FITC^+^/PI^−^) represents early apoptotic cells. Data are given as means ± SD and are representative of three separate experiments. *p<0.01 versus control cells; #*p*<0.01 versus HF group; ^$^ *p*<0.01 versus 24 h FP group.

The TUNEL signal, as an apoptosis marker, appeared as a bluish-violet color, while the denser nuclei frequently moved towards the cell periphery. The percentage of apoptotic cells (% of apoptosis) in the control group was 7%, and this was increased to 22% in the HF group and up to 38% in the FP group after 48 h. There were a significant differences in apoptosis between the treated and control groups (p<0.05), as seen in Figure 3A and C. These results indicate that both FP and HF are potent inducers of apoptosis, but the effect of FP is stronger than that of HF.

To determine if cell death was accompanied by the development of an apoptotic or necrotic process, we further analyzed and quantified the phenotypic changes in apoptotic cells by double staining HeLa cells with Annexin V-FITC and PI. Cell apoptosis increased significantly after treatment with 10, 20, 40 and 80 µM FP/HF for various durations, compared to the control group (Figure 3B, D and E). After treatment for 24 h, >40 µM FP could increase cell apoptosis, and 80 µM FP indirectly resulted in 89% apoptosis, whereas 80 µM HF only induced 12% apoptosis. In cells treated with 20 µM FP or HF for 48, 72 and 96 h, apoptosis induction was increased at 72 h, suggesting later stages of apoptosis in culture (Figure 3B and E). As expected, cell death in the control group remained below 7%. These results are consistent with the results of the TUNEL method, further showing that HF and FP could induce apoptotic cell death in cervical cancer cells.

### Effects of FP and HF on expression of PCNA in Hela cells

**Figure 4:**
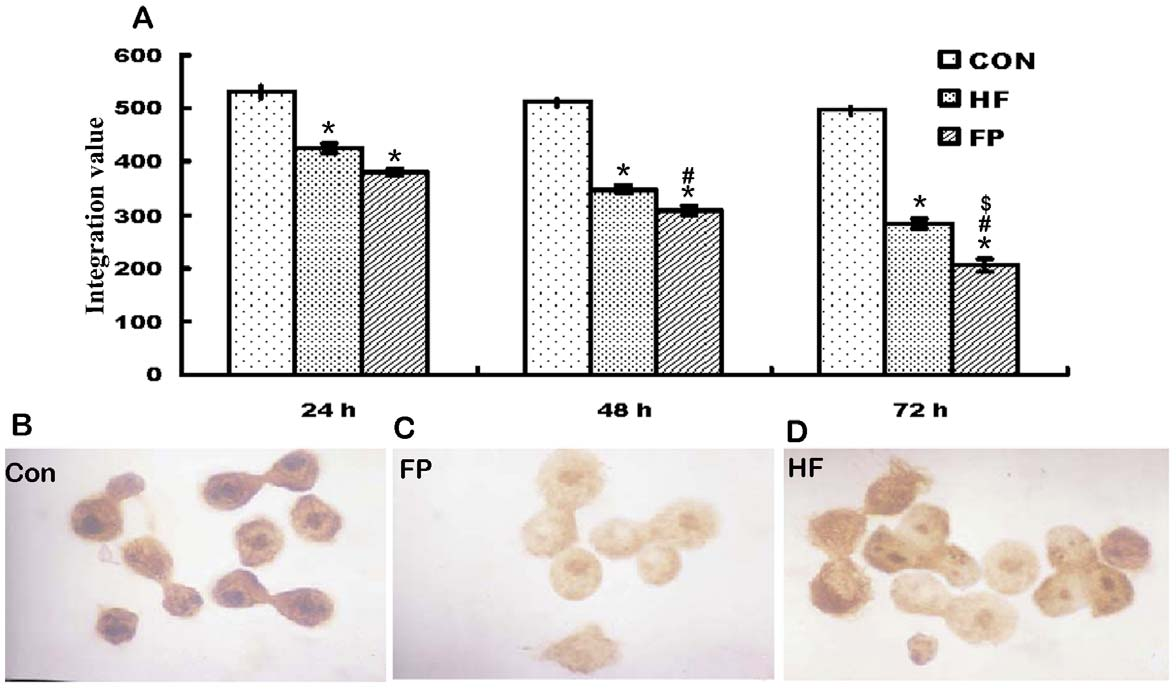
PCNA-immunoreactivity (IR) was detected by brownish staining in HeLa cells after treatment for 48 h, 1,000×. A) PCNA-IR integration values (IV) of each group after treatment for 24, 48 and 72 h were analyzed as described in Materials and Methods. B) PCNA-IR was localized in the nuclei in the control group. C) PCNA-IR was attenuated in the FP group. D) PCNA-IR was attenuated and inactive PCNA-IR was also located in the cytoplasm in the HF group. Values are given as means ± SE. *p<0.001 versus control cells; #*p*<0.01 versus HF group; ^$^ *p*<0.01 versus 24 h FP group.

PCNA immunoreactivity (IR), represented by brownish-colored granules, was located mainly in the nuclei. Inactivated PCNA was located primarily in the cytoplasm, and translocated to the nuclei once activated. PCNA-IR intensities evaluated as the integration value (IV) after 48 h were 308 in the FP group and 348 in the HF group, which were significantly higher than the control group (512) (*p*<0.05) (Figure 4). These results further showed that FP inhibited proliferation more intensively than HF, as demonstrated by the PCNA expression signal intensity in HeLa cells.

### Effects of FP and HF on expression of p21/Waf1, caspase-3 and poly (ADP-ribose) polymerase (PARP)

**Figure 5:**
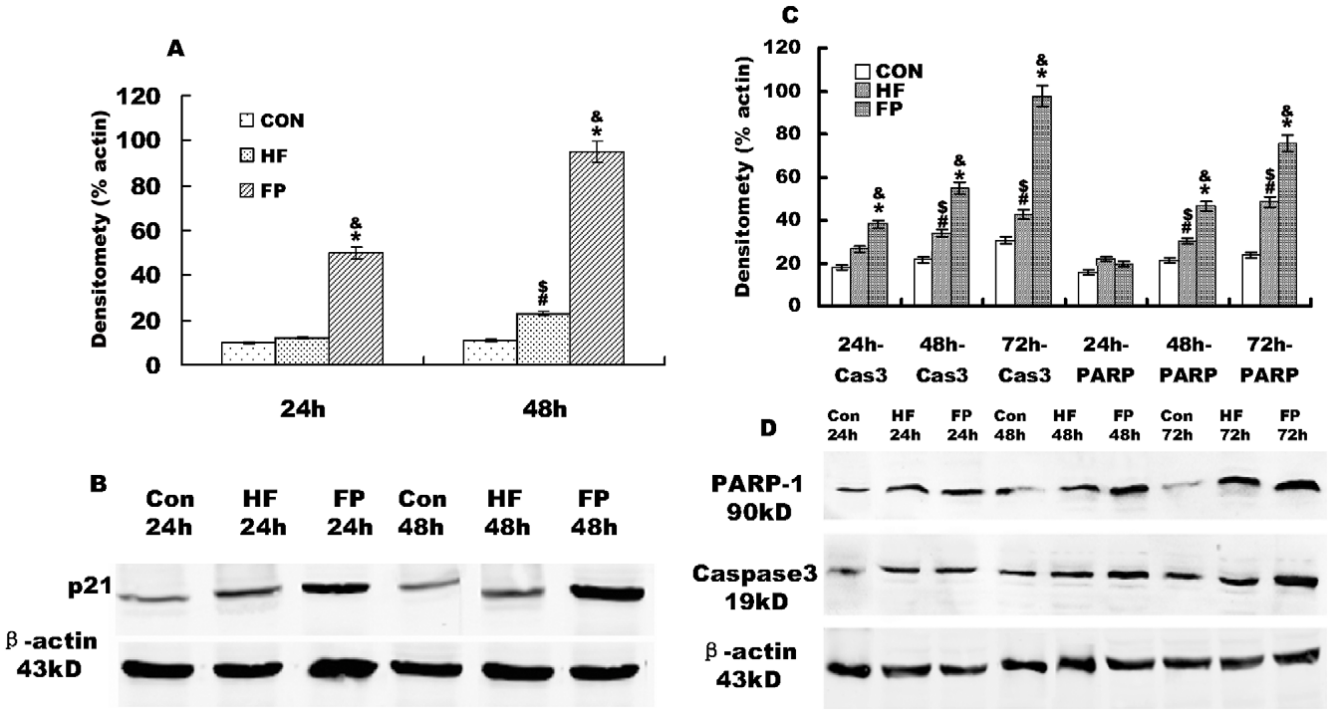
Effects of HF and FP on p21, caspase-3, and PARP in Hela cells by western blotting analyses. A) Histogram representing p21. B) Representative western blots of p21/Waf1 in FP, HF and control groups after 24 and 48 h. C) Histogram representing cleaved caspase-3 (19 kD) and cleaved PARP-1 (90 kD). D) Representative western blots of cleaved caspase-3 and cleaved PARP-I in the FP, HF and control groups after 24, 48 and 72 h. Total extracts obtained from HeLa cells without HF or FP treatment (Con), with 20 µM HF treatment (HF) and with 20 µM FP treatment (FP) were subjected to western blot analysis as described in Materials and Methods. The densitometry measurements represent the amount of p21, caspase 3, and PARP1 relative to β-actin and are given as means from triplicate experiments expressed as % of β-actin ± SE. β-actin was used as a loading control. *p<0.001 versus control cells; #*p*<0.01 versus control group; ^$^ *p*<0.01 versus FP group; ^&^ *p*<0.01 versus HF group.

To determine the mechanisms responsible for cell cycle arrest and apoptosis by HF/FP, the protein expression levels of p21/Waf1 (a cyclin-dependent kinase (CDK) inhibitor), and the apoptosis-related proteins cleaved caspase-3 and PARP-1 were examined using western blotting analyses. The expression levels of p21/Waf1 in the FP group were markedly increased in a time-dependent fashion, compared to the control group (Figure 5A and B), with more prominent expression after 48 h than after 24 h. p21/Waf1 expression in the FP group started to increase at 24 h, and became especially obvious at 48 h, whereas its expression in the HF group did not become apparent until 48 h, compared to the control group. Changes in p21/Waf1 expression in the FP treatment group were more marked (7-fold) in Hela cells, compared to that in the HF treatment group during the same period. This suggests that the up-regulation of p21/Waf1 might be related to the cell cycle arrest induced by HF and FP.

Levels of the apoptotic proteins, cleaved PARP-1 (90 kD) and caspase-3 (19 kD), were significantly elevated in FP- and HF-cultured cells, compared to untreated control cells (Figure 5 C and D). Cleaved caspase-3 started to increase at 24 h after FP treatment, and became especially obvious at 48 h and 72 h. While cleaved PARP-1 started to increase at 48 h after FP treatment, and became especially obvious at 72 h. In contrast, cleaved caspase-3 and PARP-1 became apparent after 72 h in the HF group. There were significant differences in cleaved- caspase 3/PARP-1 between the FP and HF groups. These results suggest that FP and HF induced HeLa cell injury or death by activating an apoptotic pathway involving impaired nuclear function and cellular homeostasis.

### Effects of FP and HF on cAMP concentrations in Hela cells and PDE inhibition activity *in vitro*

**Figure 6:**
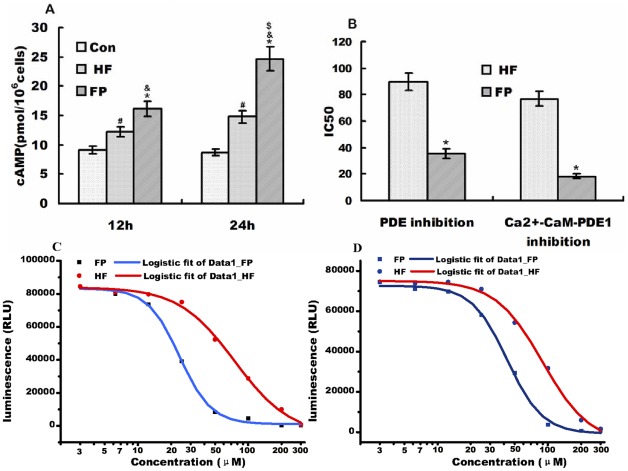
cAMP levels in Hela cells in each group and PDE inhibition activities *in vitro*. A) cAMP concentrations in Hela cells in each group at 12 and 24 h. CON, control; HF, 20 µM HF group; FP, 20 µM FP group. B) Histogram representing inhibitory effects of FP and HP on PDE and CaM-activated PDE1 activity. C) Inhibitory effects of FP and HP on CaM-activated PDE1 activity. D) Inhibitory effects of FP and HP on PDE activity from bovine brain. Values are given as mean ± SE; RLU (relative light units) on the y axes of panels C and D is positively related with CaM-activated PDE1 or PDE activities. The IC_50_ was calculated by using a sigmoidal dose-response (variable slope) equation analysis. ^&^ *p*<0.01 versus control group; *p<0.001 versus HF group; ^#^ *p*<0.01 versus control group; ^$^ *p*<0.01 versus 24 h FP group.

Hela cells were pre-incubated in medium for 24 h and then incubated with 20 µM HF or FP at 37°C for a further 12 or 24 h. The concentrations of cAMP in the cells were measured in the various experimental groups (Figure 6A). The cAMP concentrations were significantly increased to 12.2±0.67 pmol for HF and 16.2±0.87 pmol for FP at 12 h, and 15.2±0.83 (HF) and 24.6±1.68 (FP) pmol at 24 h, compared to control cells (9.1±0.78 pmol). These results represent increases of 134% and 178% at 12 h, and 167% and 270% at 24 h, respectively, which were significantly different from the levels in control cells (*p*<0.05, *p*<0.01). FP had a more significant effect on cAMP concentrations than HF.

Both HF and FP inhibited the activation of PDE or CaM-activated PDE1 from bovine brain in concentration-dependent manners (Figure 6 B, C and D). FP inhibited PDE over a 5–100 µM concentration range. CaM-activated PDE1 was preferentially inhibited with an IC_50_ value of 22.3 µM, which was significantly (two-fold) lower than the IC_50_ (43.5 µM) necessary for inhibition of basal PDE1, indicating that FP could also interact with CaM. In contrast, HF displayed lower inhibitory activities against both basal PDE and CaM-activated PDE, with IC_50_ values of 89.6 and 76.7 µM, respectively.

### Stoichiometry of FP and CaM of ESI-MS

**Figure 7:**
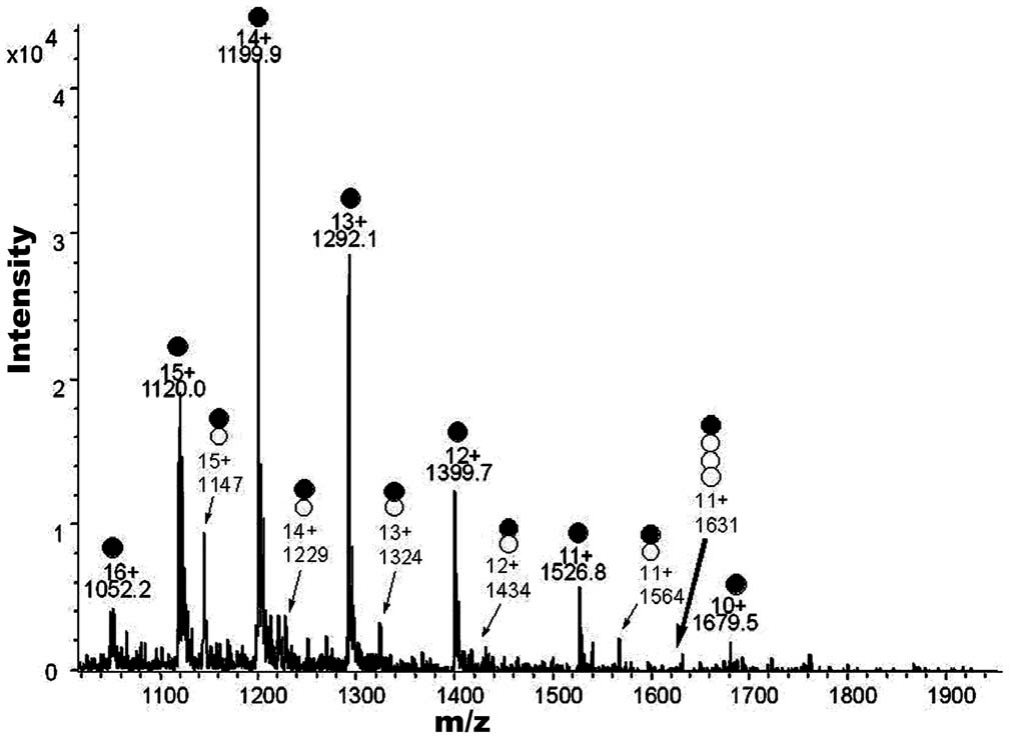
Electrospray ionization mass spectrum of CaM-Ca^2+^ with FP. The solution was prepared by mixing equal volumes of a 0.4 mM methanol solution of FP and 0.04 mM CaM-Ca^2+^. •Multiply-charged ion peaks of CaM-Ca^2+^;•○ multiply-charged ion peaks of CaM-Ca^2+^-FP complex.

Figure 7 shows the electrospray ionization (ESI) spectrum obtained from a mixed solution of CaM-Ca^2+^ and FP. The CaM charge-state distribution comprised several charge states ranging from 16^+^ to 10^+^, with 14^+^ being the most intense. The CaM mass derived from these peaks 1052.2 (CaM+16H)^16+^, 1120.0 (CaM+15H)^15+^, 1199.9 (CaM+14H)^14+^, 1292.1 (CaM+13H)^13+^, 1399.7 (CaM+12H)^12+^, 1536.8 (CaM+11H)^11+^ and 1679.5 (CaM+10H)^10+^ was 16,784 kD. Besides the expected multiple protonated molecule ions, the mass spectrum revealed several groups of new protonated ions, corresponding to several kinds of highly charged multiple adducts, e.g., ions at m/z 1147.5, 1229.4, 1323.9, 1434.2, and 1564.5, corresponding to (Ca^2+^-CaM+FP)^15+^, (Ca^2+^-CaM+FP)^14+^, (Ca^2+^-CaM+FP)^13+^, (Ca^2+^-CaM+FP)^12+^ and (Ca^2+^-CaM+FP)^11+^. The CaM+Ca^2+^+FP mass derived from these peaks was 17,198 kD (16,784+40+374). For comparison, 0.4 mM HF was also mixed with 0.04 mM CaM-Ca^2+^ and infused to ESI, but no corresponding noncovalent CaM+Ca^2+^+FP complex was detected, despite the use of various parameters. The results of these experiments indicate that FP (phosphorylated HF) was able to form a noncovalent complex with CaM+Ca^2+^ more easily than nonphosphorylated HF, suggesting that phosphorylation of esters of HF could enhance their interaction with proteins.

### Effects of FP on emission spectra of CaM-Ca2+-PDE system

**Figure 8:**
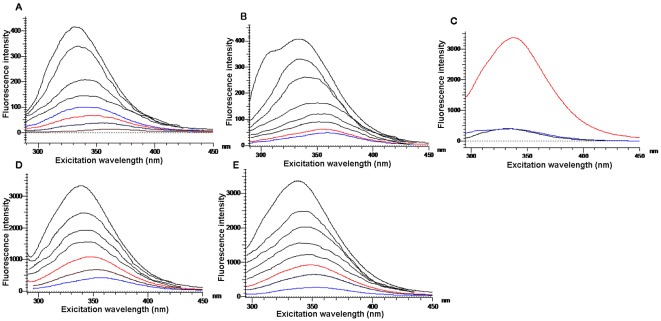
Effect of PE on fluorescence spectra in different protein solutions. A) Effect of FP on fluorescence spectra of CaM. B) Effect of FP on fluorescence spectra of PDE; C) Enhanced fluorescence spectra of CaM and PDE in presence of Ca^2+^. D) Effect of FP on fluorescence spectra of CaM-Ca^2+^-PDE at 37°C. E) Effect of FP on fluorescence spectra of CaM-Ca^2+^-PDE at 25°C.

The interaction between CaM and FP is shown in Figure 8A. When the FP concentration was increased, the emission peak of CaM decreased in each case, and the maximum emission wavelength increased from 330 to 350 nm.

**Figure 9:**
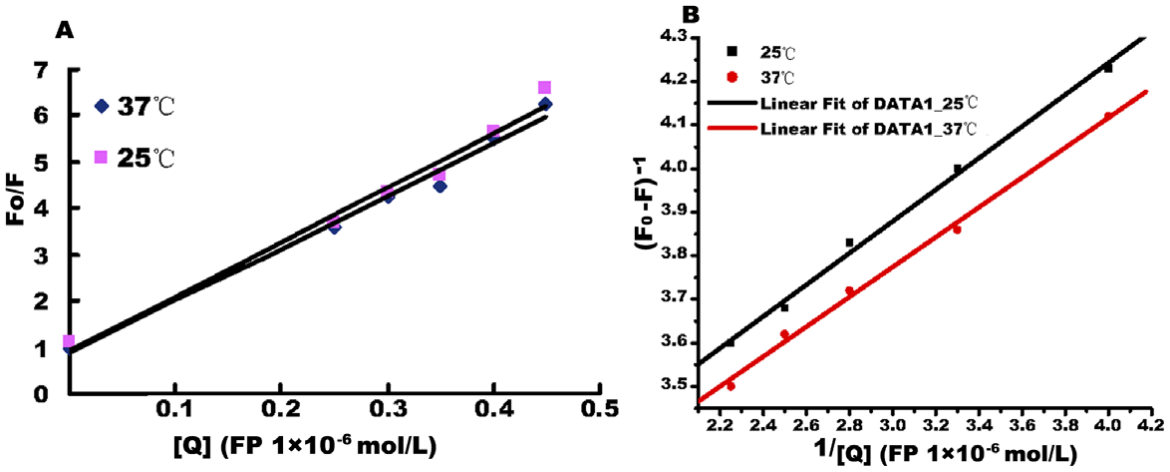
Calculation of binding constants. A) Stern-Volmer plots of FP and CaM-Ca^2+^-PDE system at different temperature (25°C and 37°C). B) Line weaver-Burk plots of FP versus CaM-Ca^2+^-PDE at 25°C and 37°C; *k*_25°C_ = 6.24×10^4^ M^−1^ and *k*_37°C_ = 4.8×10^4^ M^−1^, *K* represents binding constant.

The interaction between PDE and FP is shown in Figure 8B. The emission peak of PDE also decreased, accompanied by an increase in FP concentration, while the maximum emission wavelength increased from 335 to 360 nm. When equal concentrations of CaM and PDE were mixed with Ca^2+^, the emission intensity of the CaM-Ca^2+^-PDE system (λ = 344 nm) increased significantly from 400 to 3,400 (Figure 8C), indicating that the CaM-Ca^2+^-PDE enzyme system exhibited a strong intermolecular interaction. Figure 8D shows the emission spectra of the CaM-Ca^2+^-PDE system with increasing concentrations of FP; the emission intensity apparently decreased as the FP concentration increased, and the maximum emission wavelength increased simultaneously by 20 nm (from 340 to 360 nm). In contrast, the emission intensity of the CaM-Ca^2+^-PDE system decreased slightly as the HF concentration increased. According to the classical Stern-Volmer equation [12]:
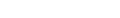
where *F_0_* is the emission intensity in the absence of quencher, *F* is the emission intensity in the presence of quencher, *Kq* is the quenching constant and [Q] is the quencher concentration. The shape of the Stern-Volmer plots can be used to characterize the quenching as either predominantly dynamic or static. Plots of F_0_/F versus [Q] appear to be linear and Kq depends on temperature. The emission quenching on addition of FP to the CaM-Ca^2+^-PDE system at 25°C is shown in Figure 8E. *F_0_/F* – [Q] lines at 25°C and 37°C, respectively, are shown in Figure 9A. The experiments demonstrated that a higher temperature was associated with a lower the slope of the quenching curve for the CaM-Ca^2+^-PDE system in presence of different amounts of FP. The combination of FP with CaM-Ca^2+^-PDE was a single static quenching process. The quenching data were therefore analyzed according to the equations
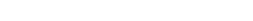
Line weaver-Burk plots are shown in Figure 9B. The linearly-dependent coefficients were 0.99 and 0.998. According to this equation, the binding constants at different temperatures could be calculated as k_37°C_ = 4.8×10^4^ L/mol, k_25°C_ = 6.24×10^4^ L/mol, respectively. These results showed that FP, but not HF, formed noncovalent complexes and showed high binding affinity with CaM-Ca^2+^-PDE.

## Discussion

Most nontoxic dietary flavonoids are known to behave as general cell growth inhibitors in many kinds of cultured human cancer cell lines [13], [14]. Many flavonoids can perturb cell cycle progression and induce G1 or G2/M cell cycle arrest, which is a fundamental activity in the process of cell proliferation [15], [16]. The present results demonstrated that both HF and FP were able to significantly inhibit proliferation in human cervical cancer HeLa cells in dose- and time-dependent manners, with FP (IC_50_ value, 18.5 µM at 72 h) having a much greater effect than HF (IC_50_ value, 32.1 µM at 72 h). The present study also demonstrated a novel characteristic of FP in terms of the cell cycle, by observing that cell populations showed greatly decreased S phases and increased G0/G1 and G2/M phases compared to controls, indicating that FP controlled cell cycle progression at both the G1/S and G2/M check-point transitions, whereas HF only arrested cell cycle progression at the G1/S check-point. The dose- and time-dependent effects on the cell cycle could contribute to the anti-proliferative effects of FP and HF.

Cell cycle arrest can lead to induction of apoptosis, and agents that modulate apoptosis affect the steady-state cell population, which is a useful means of increasing tumor-cell apoptosis for cancer therapy [17]. TUNEL labeling detected cells with typical apoptotic features after HF and FP treatment. The phenotypic changes characteristic of apoptosis were confirmed by cytometric analysis using double staining with FITC-Annexin V and PI. The levels of apoptosis induced by HF (<80 µM) or FP (<40 µM) treatment for 24 h indicated that HF mainly arrested cell cycle progression, not apoptosis, while FP arrested the cell cycle and induced apoptosis even in the early stages. The levels of apoptosis after treatment with 20 µM FP or HF for 48, 72 and 96 h demonstrated increasing cell death of Hela cells after HF and FP uptake for 72 h, which represents a later stage of apoptosis. Thus, although both FP and HF could induce HeLa cell apoptosis, FP was more potent than HF in terms of activating apoptosis, indicating that FP may represent a potential anticancer agent for use in human cervical cancer therapy. Cell growth is controlled by the balance between proliferation and programmed cell death (apoptosis). In order to further understand the molecular mechanisms responsible for the anticancer activities of HF and FP, the expression levels p21/Waf1, PCNA and apoptosis-related proteins were examined.

p21/Waf1 inhibits CDK activities and prevents cell cycle progression [18], [19]. The development of sporadic tumors is generally associated with reduced expression of p21/Waf1 [20]. Moreover, increased expression of p21/Waf1 has been demonstrated to inhibit proliferation and promote apoptosis of malignant cells *in vitro* and *in vivo* [21], [22]. PCNA is markedly expressed in proliferating cells and in most malignant tumor cells, and is therefore used as a proliferative/malignancy biomarker in cancer [23], [24]. Cleaved caspase-3 has been verified as an activated form of caspase-3 that acts as a lethal protease at the most distal stage of the apoptotic pathway [25]. At the onset of apoptosis, caspase-3 proteolytically cleaves PARP, which is a nuclear DNA-binding zinc-finger protein that influences DNA repair, DNA replication, modulation of chromatin structure, and apoptosis [26].

We investigated the involvements of p21/Waf1 and PCNA proteins in the antiproliferative effects of HF- and FP in HeLa cells. FP induced cell cycle arrest in both G0/G1 and G2/M phases as shown by up-regulation of p21 and down-regulation of PCNA in HeLa cells. Enhanced expression of cleaved caspase-3 and PARP-1 (90 kD) in FP- or HF-treated HeLa cells at 48 h provided further evidence for promotion of apoptosis by FP and HF. However, the levels of cleaved PARP-1 were lower than those of cleaved caspase-3 after FP and HF treatment, indicating that activated caspase-3 may activate other downstream caspase family effectors. FP is characterized by the presence of a 7-hydroxyflavone basic ring skeleton, with an extra phosphate ester substitution. This structural difference is related to its mechanism of action and potency. Together, FP-induced apoptosis and proliferation inhibition contribute directly to up-regulation of p21/Waf1, decreased PCNA, and increased cleavage of caspase-3 and PARP-1. In contrast, 7-hydroxyflavones lacking this phosphate ester substitution mediated cell cycle arrest in G0/G1 phase and showed antiproliferative activity consistent with inhibition of PCNA activity. The induction of p21/Waf1 by HF might be responsible for cell-cycle arrest but not apoptosis (elevated p21 vs. no obvious apoptosis for HF at 48 h). These results suggest that FP and HF might induce growth inhibition and apoptosis by different mechanisms.

cAMP signaling pathways are known to provide major routes for controlling cellular function [27], [28]. In an *in vitro* experiment, cAMP was considered to exert a growth-inhibitory action on SKOV cells via induction of p21 expression and cell cycle arrest, with accumulation of cells in the G0/G1 fraction of the cell cycle [29]. In the present study, the FP-mediated activation of p21/Waf1 in Hela cells elicited a profound increase in cAMP concentrations, which appear to inhibit cell growth through induction of p21 expression and inhibition of PCNA and cell cycle progression in G1 or G2/M. cAMP inactivation is known to occur through hydrolysis to 5′-AMP, which is achieved by cAMP PDE activity [30], [31]. Within the PDE enzyme family, PDE1 activities are stimulated by Ca^2+^ and CaM, and can specifically control cAMP degradation within the cell regulatory system. In addition, these isoenzymes may be inhibited by several flavonoids [32]. As previously reported for a number of non-selective PDE inhibitors, the current results showed that FP preferentially inhibited CaM-activated PDE1 (which hydrolyzes cAMP) isolated from bovine brain, with an IC_50_ value of 22.3 µM, which was significantly lower than the IC_50_ (43.5 µM) necessary for inhibition of basal PDE, indicating that FP also interacts with CaM. Both HF and FP may thus act as PDE inhibitors with different activities, resulting in increased levels of cyclic nucleotides. It is thus likely that the effect of FP on p21 regulation may be associated with an increase in intracellular cyclic nucleotide levels (via PDE inhibition). FP showed greater potency than HF in most of the current experiments, indicating that the presence of a phosphate ester in the chemical structure of FP (7-position) increases its pharmacological activity by producing a spatial conformation that favors interaction with PDE and other potential cellular targets.

Comprehensive studies on the nature of the interactions between small molecules and proteins are of fundamental importance in the biomedical and pharmaceutical sciences. In this context, we extended the scope of this study to examine the interaction between FP and CaM-Ca^2+^-PDE1 using fluorescence spectrometry combined with ESI-MS. A new noncovalent complex (FP-CaM-Ca^2+^) analyzed by ESI-MS further verified the stronger binding affinity of FP with CaM-Ca^2+^, compared to HF. Furthermore, the FP-CaM-Ca^2+^ noncovalent complex was likely to influence PDE1 species activities via specific CaM-Ca^2+^ stimulation. The fluorescence intensity of a mixed solution of CaM-Ca^2+^ and PDE *in vitro* was also significantly elevated compared to either CaM-Ca^2+^ or PDE1 alone, confirming that specific induction by Ca^2+^/CaM stimulated PDE1 activity. The effects of FP on the emission spectra of the CaM-Ca^2+^-PDE protein-enzyme complex system demonstrated an enhanced molecular interaction between FP and the CaM-Ca^2+^-PDE enzyme complex system.

Determination of the FP and CaM-Ca^2+^-PDE1 binding constants provided information on the binding sites and the main acting forces in the studied complexes. The binding effects detected by fluorescence and ESI-MS assays were in agreement with the results of PDE activity, despite the different principles of the methods. The results obtained could facilitate our understanding of the physiological effects by focusing on the structural features. Our previous study showed that the individual biological potency of the phosphate ester of chrysin was related at least partly to its phosporylated structure [11]. Phosphorylated flavones might thus combine with the active site of PDEs by H-bond interactions, preventing the CaM-Ca^2+^-PDE enzyme complex from effectively hydrolyzing cAMP.

To summarize, this study demonstrated that FP has clear anticancer effects in Hela cells, probably mediated by an increase in cytosolic cAMP via inhibition of PDE. FP exerts growth inhibitory and apoptosis-inducing actions through induction of p21, caspase-3 and PARP-1, and down-regulation of PCNA associated with cell cycle arrest. In contrast, HF has lower activity against HeLa tumor cells under the same conditions. These observations clearly demonstrate the importance of phosphorylated flavonoids in biological processes, and suggest that FP might have promising potential as a new anti-cancer drug.

## Materials and Methods

### Test compounds

**Figure 10:**
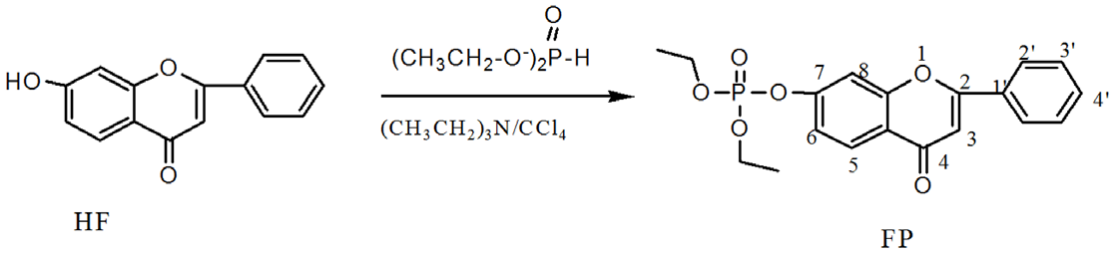
Scheme of diethyl flavon7-yl phosphate (FP) synthesized by a simplified Atheron-Todd reaction.

HF (7-Hydroxy-flavone, C_15_H_10_O_3_,Mr: 238.24) was purchased from Sigma (St. Louis, MO, USA), and FP was synthesized by a simplified Atheron-Todd reaction (Figure 10).

### MTS assay

The test compounds (HF and FP) were dissolved in dimethyl sulfoxide (DMSO) ≤0.1%. HeLa cells (human cervical carcinoma, Shanghai Institutes of Biological Sciences) were cultured in DMEM (Gibco, Invitrogen, USA) supplemented with 10% fetal bovine serum (Gibco). Cell viability was measured in the presence or absence of HF or FP using a cell viability assay, as previously described [33]. The cells were harvested in the log phase of growth and inoculated onto 96-well plates at a final concentration of 3×10^3^ cells per well. After 24 h of incubation at 37°C under 5% CO_2_, the cell cultures were treated with FP/HF at 5, 10, 20, 40, 80 and 160 µM. [3-(4,5-dimethylthiazol-2-yl)-5-(3-carboxymethoxyphenyl)-2(4-sulfophenyl)-2H-tetrazolium/phenazine methosulfate (MTS/PMS; Promega, Madison, WI, USA) 20 µl was added to each well, and the absorbance at 490 nm was measured according to the manufacturer's recommendations. The mean value was obtained from four replicate wells after exposure to the agents for 24, 48, 72 and 96 h, respectively. A control group treated with no any agents was performed simultaneously. The growth inhibition ratio was calculated according to the following equation: inhibition ratio (%) = (control group−treated group)/control group×100%.

### Preparation of specimens

20 µM HF and FP were added to HeLa cell cultures for 24, 48 and 72 h. TUNEL, PCNA immunocytochemistry tests and western blotting (p21/Waf1, caspase-3 and PARP1) were performed, based on the above MTS assay. Experimental and control specimens for TUNEL and immunocytochemistry assays were fixed on slides with 4% paraformaldehyde for 20 min at room temperature, followed by washing in phosphate-buffered saline (PBS), and storage at −20°C.

### Cell-cycle analysis

HeLa cells were seeded at a density of 1×10^5^ onto 6-well plates and incubated for 24, 48, 72 and 96 h in the absence or presence of the test compounds (10, 20, 40 and 80 µM concentrations after reaching 70% confluency. The cells were washed twice with PBS, then harvested by trypsinization and washed twice with PBS. Then pellets were resuspended and fixed in 70% alcohol for 1 h at 4°C. The suspension was then centrifuged in a microfuge for 5 min at 800 rpm, and the pellet was resuspended in PBS containing PI (50 µg/ml), Triton X-100 (0.1%, v/v) and RNase A (100 µg/ml). After 30 min incubation, the cell cycle distribution was examined using a FACScano II SE flow cytometer with Modfit software (Becton Dickinson, San Jose, CA).

### TUNEL method for detection of apoptosis

TUNEL analysis to detect apoptosis was performed as described previously, with 1 µl terminal dUTP-transferase (TdT, Promega, Madison, WI, USA) and 1 µl biotin-16-dUTP (Promega) [11]. Positive bluish-violet signals were visualized using streptavidin- alkaline phosphatase reaction with 5-bromo-4-chloro-3-indolyl-phosphate and nitroblue tetrazolium (BCIP-NBT, Promega) as the substrate. TdT was omitted from the buffer in the negative control specimen. The percentage of apoptosis (% of apoptosis) according to the positive signal was calculated for each slide, in which ten fields of view were selected under an oil-emersion microscope (Nikon, TE2000-U, Japan). Apoptosis (%) = (number of apoptosis cells/total number of tumor cells)×100.

### Annexin V-FITC/PI double staining for apoptosis by flow cytometry

HeLa cells were seeded at a density of 5×10^5^ onto 6-well plates and incubated for 24, 48, 72 and 96 h in the presence or absence of the test compounds (10, 20, 40 and 80 µM). Apoptosis assay was performed with an Apoptosis Assay Kit (Baosai Biology, Beijing, China) using a flow cytometer, following the manufacturer's instructions. Briefly, cells were trypsinized and washed with PBS. The pellets were resuspended in PBS to a concentration of 5×10^5^ cells. The cells were then incubated with 10 µl FITC-Annexin-V dissolved in 300 µl binding buffer in the dark at room temperature for 15 min. The pellets were stained with 5 µl PI in 200 µl binding buffer. Emission wavelengths of 518 nm (Annexin V-FITC) and 617 nm (PI) were used to identify the populations of viable cells (Annexin V^−^PI^−^), early apoptotic cells (Annexin V^+^PI^−^), necrotic cells (Annexin V^−^PI^+^), and late apoptotic cells (Annexin V^+^PI^+^). The collected events per sample were 10,000. Apoptotic percentage (AP %) = (upper right quadrant (UR)+lower right quadrant (LR)) %.

### Immunocytochemistry

Paraformaldehyde-fixed slides were treated with 0.5% Triton X-100/PBS solution for 30 min followed by 3% H_2_O_2_ for 5 min. The slides were heat-renatured in 0.05 mM citric acid buffer (pH 6.0 for antigen retrieval), and blocked with 1∶10 diluted normal goat serum in PBS for 30 min. After removal of excess serum, slides were incubated overnight at 4°C with monoclonal antibody against PCNA (Zymed, Invitrogen, USA) freshly diluted at 1∶100, followed by incubation with biotin-labeled goat anti-mouse serum and streptavidin conjugated peroxidase at 37°C for 20 min. 3, 3′diaminobenzidine 0.05% and 0.01% H_2_O_2_ were used as substrates to develop a brownish positive IR signal. PBS was substituted for the specific primary antibody in the negative control. Slides were examined and 200 cells were counted in ten different fields of view by two independent, blinded observers, using an oil-emersion microscope. The IV of PCNA-IR was based on a previously reported method [34], [35], and modified as follows: 0 = negative (−, no appreciable staining in cells); 1 = weak positive (+, readily-appreciable brown staining distinctly marking the tumor cell cytoplasm and/or nucleus); 2 = medium positive (++, dark brown staining in tumor cells obscuring the cytoplasm and/or nucleus); 3 = strong positive (+++, very strong staining of nucleus and/or cytoplasm). The IV of PCNA-IR was scored semi-quantitatively by multiplying the number of positive cells (0–200) by the staining intensity (grade 1–3), thus producing a score of 0–600.

### Western blotting analysis of caspase-3, PARP-1 and p21/waf1 proteins in Hela cells

HeLa cells were seeded in 24-well plates (5×10^4^/well) and cultured for 24 h. HF or FP 20 µM were added to the cells for a further 24 or 48 h. Extracts from each group cells were prepared and western immunoblotting analysis was performed as follows: The cells were washed twice with PBS, scraped from the culture dishes, and treated for 20 min on ice with lysis buffer containing 1 mM EGTA, 1 mM EDTA, 150 mM NaCl, 1% Triton X-100, 2.5 mM sodium pyrophosphate, 1 mM phenylmethyl-sulfonyl fluoride, 1 mM NaVO_4_ and 1 mg/ml leupeptin, 1 mg/ml aprotinin and 20 mM HEPES-KOH (pH 7.9). Cell lysates were centrifuged at 13,000 rpm for 15 min at 4°C. Total protein concentrations were determined and equal amounts of proteins in each group were then separated by 12% sodium dodecyl sulfate-polyacrylamide gel electrophoresis. The gels were transferred to a nitrocellulose membrane (Amersham, GE Heathcare, USA) using a semi-dry electroblotting system (Bio-Rad, USA). The membranes were blocked with 3% (w/v) bovine serum albumin in PBS with 0.2% (v/v) Tween-20 (PBST) for 1 h, and then incubated with the primary mouse monoclonal antibody (anti-caspase3 and anti-PARP-1, Santa Cruz Biotechnology, Santa Cruz, CA, USA, 1∶200), mouse monoclonal antibody (against p21^Waf1^, Santa Cruz, 1∶200), and β-actin monoclonal antibodies (1∶1,000; Santa Cruz) at 4°C overnight with rocking. Membranes were rinsed three times with PBST, and incubated with secondary goat anti-mouse IgG conjugated with IRDye700 fluorescence in (1∶10,000 dilution; LI-COR, USA) for 1 h at room temperature. Membranes were rinsed twice with PBST and once with PBS. Subsequent analysis was performed using a Li-COR Odyssey system and quantified using Odyssey infrared imaging software, with β-actin as a loading control.

### RIA examination of cAMP in cells

HeLa cells were seeded at a density of 3×10^4^ onto 24-well plates and incubated for 12 or 24 h in the presence or absence of the test compounds (20 µM). Cells were washed three times with PBS and then freeze-thawed twice with 0.5 ml cold HCl (50 mM) at −80°C. Cell lysates were centrifuged at 13,000 *g* for 10 min at 4°C. The supernatants were freeze-dried and stored at −80°C. The cAMP concentration of each sample was measured using commercial RIA kits (Shanghai University of Traditional Chinese Medicine, Shanghai, China). The minimum detection limit for cAMP was 0.1 pmol/ml for nonacetylated samples; cross-reaction with cGMP was less than 0.001%. In brief, duplicate aliquots of samples (100 µl) and 100 µl of each point of cAMP standard curve (20,10, 5, 2.5, and 1.25 pmol/ml) were dispensed into conical tubes containing 100 µl of assay buffer, then incubated with 100 µl of ^125^I-cAMP (25,000 cpm) and 100 µl of diluted primary antibody (rabbit anti-cAMP). The initial dilution of the antiserum was 1∶500. The maximum binding (B_0_) was determined by replacing standard cAMP by 100 µl of assay buffer. The nonspecific binding (NSB) tubes contained 200 µl of assay buffer and 100 µl of ^125^I-cAMP. Total count tubes (Tc) contained 100 µl of ^125^I-cAMP. The following day, bound and free fractions were separated by adding 100 µl of second-antibody solution (0.05% v∶v normal rabbit serum, 0.45% v∶v sheep anti-rabbit anti serum,0.4% w∶v BSA, 0.05% w∶v microcrystalline cellulose, 0.5%w∶v polyethylene glycol 6000 in phosphate buffer) to all except the Tc tubes, followed by further incubation (1.5 h) at room temperature. The tubes were then washed with 2 ml of phosphate-BSA-Tween 20™ buffer and centrifuged at 2,500 *g* at 4°C for 30 min. The supernatant was discarded and the pellet was washed again. The radioactivity was measured in a gamma counter with an efficiency of 75%.

### Determination of PDE activity

PDE activity was measured using the PDE-Glo™ Assay (Promega, Madison, WI, USA), according to the manufacturer's instructions. HF and FP were serially titrated manually at 1∶2, and added to the assay plate containing bovine brain PDE (Sigma-Aldrich Cat.# P9529) or CaM-activated PDE, in which 2 U CaM (Sigma-Aldrich Cat.# P4874) were incubated with 0.015 U PDE and 0.03 mM Ca^2+^ for 30 min, and pre-incubated for 5 min before substrate addition. cAMP substrate 2 µM was added to each well for 5 min. Luminescence was recorded using a GloMax®-Multi Microplate Reader (Promega). Value was expressed as a relative light units (RLU). The IC_50_ values were determined by non-linear regression analysis by fitting to hyperbolic inhibition. The maximal final concentration of DMSO in the control samples had no effect on PDE activity. Because of the limited solubility of the tested flavonoids, the highest concentration used in these experiments was 200 µM.

### Mass spectrometry conditions

A mass spectrometer (Bruker-Esquire 3000 Ion Trap, Bruker Daltonics, Bermen, Germany) installed with an ion-spray source working in the positive ion mode was employed. The neutralizer pressure was 12 psi, the dry gas flow rate was 9.00 l/min, and the capillary voltage was held at 4 kV. The rolling average was seven. The ion count cumulative (ICC) was set at 30,000. Dissolved samples were continuously infused into the ESI chamber at a flow rate of 4 µl/min using a 744900 syringe pump (Cole-Parmer Instrument Co., USA).

### Fluorescence spectra

The binding reactions between FP and Ca^2+^-CaM-PDE enzyme system in the aqueous phase were studied using fluorescence spectrometry. Fluorescence spectra were detected using a F4500 spectrofluorometer with an excitation wavelength of 280 nm and an emission range set between 290 nm and 450 nm. The excitation wavelength for proteins is generally about 280 nm, and this wavelength was therefore selected to study the FP and Ca^2+^-CaM-PDE enzyme system interaction. No fluorescence was emitted by FP under this excitation wavelength. The quenching experiments were repeated with different quantities of FP at 25°C and 37°C, for the same Ca^2+^-CaM-PDE enzyme concentration.

### Statistical analysis

At least three independent experiments were carried out for each variable. Data are given as mean ± SE. The statistical analysis was made with SSPS10.0. Differences (*p*<0.05), indicated by asterisks, were considered statistically significant.
